# The role of cytokines in the pathogenesis and staging of *Trypanosoma brucei rhodesiense* sleeping sickness

**DOI:** 10.1186/s13223-016-0113-5

**Published:** 2016-01-22

**Authors:** Charles D. Kato, Enock Matovu, Claire. M. Mugasa, Ann Nanteza, Vincent P. Alibu

**Affiliations:** School of Bio-security, Biotechnical & Laboratory Sciences, College of Veterinary Medicine, Animal Resources & Bio-security, Makerere University, P.O BOX 7062, Kampala, Uganda; College of Natural Sciences, Makerere University, P.O. BOX 7062, Kampala, Uganda

**Keywords:** Human African Trypanosomiasis, Cytokines, Immune response, Biomarkers, Sleeping sickness

## Abstract

Human African trypanosomiasis due to *Trypanosoma brucei rhodesiense* is invariably fatal if untreated with up to 12.3 million people at a risk of developing the disease in Sub-Saharan Africa. The disease is characterized by a wide spectrum of clinical presentation coupled with differences in disease progression and severity. While the factors determining this varied response have not been clearly characterized, inflammatory cytokines have been partially implicated as key players. In this review, we consolidate available literature on the role of specific cytokines in the pathogenesis of *T. b. rhodesiense* sleeping sickness and further discuss their potential as stage biomarkers. Such information would guide upcoming research on the immunology of sleeping sickness and further assist in the selection and evaluation of cytokines as disease stage or diagnostic biomarkers.

## Background

Human African trypanosomiasis (HAT) or sleeping sickness is caused by tsetse fly transmitted extra-cellular protozoan parasites *Trypanosoma brucei rhodesiense* (east and southern Africa) and *T. b. gambiense* (west and central Africa). It is considered that these species are clinically and epidemiological different thus requiring different therapeutic management [1]. Disease due to *T. b. rhodesiense* is classified as acute with rapid progression while *T. b. gambiense* disease is characterized as chronic [2, 3]. A reduction in the number of new HAT cases has been reported [4]. However, in endemic areas an estimated 12.3 million people living in or around national parks, forest land and large water bodies are still at a risk of acquiring *T. b. rhodesiense* disease. More so fatality cases are estimated to be higher than reported since 40 % of cases go undetected and subsequently untreated [5, 6]. *T. b. rhodesiense* HAT being a zoonotic disease and endemic in vast areas of continental/tropical Africa [4], elimination cannot be easily achieved.

Previously*, T. b. rhodesiense* HAT has been classified as an acute disease with death occurring within weeks or few months if untreated [2, 3]. Recently, a wide spectrum of clinical presentation coupled with differences in disease progression and severity was reported [7–9]. It is now believed that the disease is chronic in south east Africa and progressively more severe and acute towards the north [8, 10]. It has been demonstrated that individuals from non-endemic areas suffer a more severe disease than those in endemic countries [11, 12]. Furthermore, there seems to be differences in disease progression irrespective of whether the foci are geographically related. A study comparing early stage patients recruited in two geographically distinct areas noted median duration of illness to be longer among Malawi patients (30 days) compared to patients in Uganda (21 days) [8]. In addition, dramatic differences in disease progression and degree of neurological impairment were reported among Ugandan patients in geographically related foci [13]. Subsequently, it is not yet clear if these differences in disease progression and severity are related to the parasite diversity, to host related differences regulating immune responses or to both. However, compelling evidence suggests that cytokines might be key players in HAT inflammatory processes [8, 13, 14].

Reports from animal models and the few studies involving humans suggest that high levels of pro-inflammatory cytokines might be associated with moderate to severe neuropathy [15, 16]. Furthermore, late stage disease has been associated with elevated levels of counter-inflammatory cytokines in both HAT patients and experimental animal models [16]. Counter-inflammatory cytokines (IL-10 and IL-6) have been associated with a reduction in the severity of neuropathology, suggesting a possible protective role [15]. However, there remains controversy on the role of specific cytokines in disease progression and severity [17, 18]. In this review, we aim to consolidate available literature on the role of specific cytokines in *T. b. rhodesiense* HAT pathogenesis and to further discuss their potential as stage biomarkers. Such information would guide upcoming research in the immunology of HAT and further assist in the selection and evaluation of cytokines as stage biomarkers and/or develop novel chemotherapeutic interventions.

## Diagnosis and diagnostic problems

Since the clinical signs of HAT are non-specific, in most cases the disease is only suspected in geographical areas where it is endemic. Sleeping sickness is endemic in areas where other tropical diseases like malaria exist [7, 19, 20], making HAT an incidental finding on a blood smear meant for malaria diagnosis. Currently there is an increased bias towards the use of rapid diagnostic tests (RDTs) for the diagnosis of malaria [21]. Therefore, the advent of RDT’s for malaria will consequentially lead to reduced detection *T. b. rhodesiense* HAT as this relies on the detection of trypanosomes on blood smears. Sleeping sickness occurs in rural sub-Saharan Africa necessitating diagnostic techniques that are simple and cheap to perform [22]. A major constraint in *T. b. rhodesiense* HAT diagnosis as compared to *T. b. gambiense* HAT is the fact that no suspicion serological tests are yet available thus impairing greatly the detection of cases (both for passive and active detection). Therefore, the most feasible approach for the detection of *T. b. rhodesiense* infections is through direct microscopic observation of trypanosomes in blood, lymph node aspirates or in cerebrospinal fluids (CSF) of highly suspected individuals [23]. Unlike *T. b. gambiense* HAT, parasitemia due to *T. b. rhodesiense* is in most cases above the threshold for microscopic detection reaching values of up to 10,000 trypanosomes/ml [24]. Thick blood films prepared from a finger prick have limited sensitivity (detection limit is 5000 trypanosomes/ml) but are easy to perform with quick results [25]. In cases of low parasitemia, concentrations/enrichment methods have been used to improve sensitivity. The micro-hematocrit centrifugation technique (mHCT) has a detection limit of 500 trypanosomes/ml [26, 27] while the quantitative buffy coat technique offers an improved detection limit of <500 trypanosomes/ml [28, 29]. Mini-anion-exchange centrifugation technique [30] offers an improved sensitivity, detecting <30 trypanosomes/ml while its improvement on buffy coat goes lower than 10 trypanosomes/ml [31]. Molecular biology techniques that detect parasite nucleic acids with increased sensitivity are becoming more common. The most commonly used PCR technique in research laboratory settings has been the detection of the serum resistance antigen (SRA) to confirm the presence of *T. b. rhodesiense* [32]. The SRA gene is reported to be responsible for *T. b. rhodesiense* human serum resistance but is absent in *T. b. gambiense* sub species that is also resistant to lysis by human serum [33]. SRA is able to discriminate *T. b. rhodesiense* from other *T. b. brucei* sub species with a sensitivity equivalent to 1 trypanosome/ml [34]. Although PCR based techniques have a sensitivity of up to 96 % [35], the techniques have limited application in a field setting due to the need for a thermocycler, power supply and a cold chain for reagents. To improve on their applicability, an isothermal DNA amplification technique called loop mediated isothermal amplification (LAMP) has been developed [36, 37]. LAMP is easier to perform and requires less sophisticated equipment than conventional PCR. However, before its adoption as a diagnostic tool, further clinical validation and standardization on large cohorts is required. Other alternatives like the RNA based real-time nucleic sequence based amplification [38] and oligochromatography-PCR [39] have been developed but are yet to undergo clinical evaluation and validation.

## Disease staging

Since drug treatment for both early and late stage disease differs, it is paramount to accurately determine disease stage. Since it is impossible to stage the disease based on clinical signs, invasive examination of CSF following a lumber puncture is routinely done. Disease stage determination is vital for appropriate treatment. Patients with no trypanosomes in CSF but with a white blood cell count of ≤5 WBC/µl are classified as early stage while those with >5 WBC/µl or with trypanosomes in the CSF are considered late stage patients [23]. With inconsistences and the invasive nature of the current staging method, new stage biomarkers are being proposed [40, 41].

## Host immune responses to trypanosomes

It is now clear that sera from humans and non-human primates has the ability to kill trypanosomes [42, 43]. This ability to kill trypanosomes has been linked to the innate trypanosome lytic factors (TLF). Compelling evidence suggests that apolipoprotein LI (ApoLI) and haptoglobin-related protein (Hpr) might be crucial elements of the TLF [43, 44]. *T. b. brucei* has been shown to neutralize the trypanolytic activity of normal human serum through the serum resistance associated protein (SRA) that binds to ApoA1 [32]. Trypanosomes escape host immune recognition through antigenic variation of the membrane bound variant-specific surface glycoprotein, VSG [45]. The VSG acts as a barrier preventing components of the immune responses from accessing the underlying plasma membrane [46]. At peak parasitemia, the parasite releases VSG into circulation [47] thus inducing inflammatory responses. It has been shown that coat switching trypanosomes fail to activate B-cells until coat VSG homogeneity is achieved thus evading recognition [48]. Previously, the main mechanism involved in controlling parasitemia was through antibody production [49–51] with trypanosome specific IgM and IgG reported in the cerebral spinal fluid of late stage patients [52]. In a murine *T. b. brucei* model using B cell (µMT) and IgM-deficient mice, the role of B-cells and IgM antibodies in parasitemia control was investigated [53]. The authors demonstrated that B-cells played a critical role in peak parasitemia clearance while IgM antibodies only played a limited role. However, in another study, a T-cell-independent anti-VSG IgM response was proposed as the first line of defense against proliferating parasites [54]. However, gaps still exist on how and whether antibodies play a significant role in parasite control [55]. Evidence is building up suggesting that cytokines might be key players in HAT pathogenesis [14, 15, 56].

Most studies on cytokine dysregulations in HAT have used experimental animal models making it possible to follow immunological responses with disease progression [15, 57–61]. Compelling evidence from these studies points to a profound dysregulation in cytokine profiles as a driver for HAT pathogenesis. In general, the early stage of infection is characterized by an elevation in pro-inflammatory cytokines (IFN-γ and TNF-α) with a switch to a counter-inflammatory response in late stage infection [15, 62]. It has been demonstrated that prolonged survival to murine African trypanosomiasis might be infection stage dependent, with pro-inflammatory cytokine responses playing a critical role during early stage infection while counter-inflammatory cytokines determine survival during late stage [62]. Furthermore, cytokines in the CNS have been shown to revert to normal levels after treatment making them biomarker candidates for CNS invasion [63, 64]. The role of specific cytokines in HAT progression with emphasis on *T. b. rhodesiense* is hereby discussed. The potential roles of cytokines in sleeping sickness and gaps in cytokine research are summarized in Table 1.

**Table 1:** Potential roles and inconsistencies associated with cytokines in sleeping sickness

Cytokine	Experimental trypanosomiasis	HAT
TNF-α		
1)	Parasite growth control and extended survival [49, 66, 109]	Associated with rapid disease progression [8]
2)	Control of infection induced pathology [49, 65]	No clear role in disease pathogenesis [71, 72]
3)	Mediate development of anemia [68]	No data
4)	Involvement in neuropathology and blood brain barrier dysfunction [15, 70]	No data
IFN-γ		
1)	Enhance parasite growth [74]	Neurological response involvement [13]
2)	Parasite growth control [18, 61, 73]	No neurological response involvement [76]
3)	Mediate development of anemia [73]	No data
4)	Involvement in neuropathology and blood brain barrier dysfunction [15, 75]	No data
5)	Fever induction [60]	No data
IL-1β		
1)	Involvement in neuropathology [15, 82, 83]	No involvement in disease progression or pathology [13]
TGF-β		
1)	No data	Involvement in pathology [8] No involvement in pathology [13]
IL-6		
1)	Reduction in neuropathology [15]	No defined role despite elevation in late stage [13, 72, 76, 92, 93]
IL-10		
1)	Reduced pathology and extended survival [15, 61]	No defined role despite elevation in late stage [8, 71, 72, 76, 93]

TNF-α: Tumour necrosis factor- alpha, IFN-γ: Interferon gamma, IL-1β: Interleukin-1 beta, TGF-β: Transforming growth factor- beta, IL-6: Interleukin-6, IL-10: Interleukin-10, HAT: Human African trypanosomiasis

## Tumour necrosis factor- α (TNF-α)

Tumour necrosis factor-α (TNF-α) is a pro-inflammatory cytokine predominantly produced by macrophages and is involved in the innate immunity against intracellular pathogens. Soluble VSGs shed by live trypanosomes are thought to be the major TNF-α inducing factors [65]. To date there is still controversy on the role of TNF-α in HAT infection. Some of the studies using animal models indicate that TNF-α is likely to be a key mediator in the control of *T. brucei* infections [66–68]. In one study, a direct dose dependent lytic effect of TNF-α on purified *T. b. gambiense* parasites was reported suggesting an involvement in parasite growth control [69]. However, detrimental roles of TNF-α have also been reported. In a murine model, TNF-α knockout mice exhibited trypanosome-mediated immunopathological features such as, lymphnode associated immunosuppression and lipopolysaccharide hypersensitivity [67]. High levels of brain TNF-α were associated with moderate to severe neuropathy [15]. In a *T. b. rhodesiense* vervet monkey model, CNS TNF-α levels did not differ from controls and no association with clinical presentation was reported [60]. Furthermore, it has been demonstrated in a murine model that TNF-α might be involved in anemia associated with *T. b. rhodesiense* infections and not in *T. congolense* [68]. This demonstrates the challenges in comparing studies utilizing different trypanosome or host species. There is evidence pointing to a possible role of TNF-α in trypanosome penetration of the blood brain barrier, especially through Toll-like receptor (TLR)–MyD88–mediated signaling [70]. Human *T. b. rhodesiense* studies testing predictions from experimental animal models have started to emerge. A study comparing plasma cytokine levels between geographically isolated HAT foci reported elevated levels of TNF-α in Ugandan patients as compared to their counterparts in Malawi [8]. The author’s proposed that TNF-α might play a role in the rapid disease progression reported among Uganda patients. On the contrary, in another study in Uganda both plasma and CSF TNF-α levels remained at baseline [71], just as previously reported among *T. b. gambiense* patients [72]. From available literature for both animal models and human studies, the role of TNF-α in *T. b. rhodesiense* disease pathology remains largely unclear.

## Interferon gamma (IFN-γ)

Interferon gamma is a pro-inflammatory cytokine secreted primarily by T- and natural killer (NK) cells with a role in innate immunity and also as an inducer of the adaptive immune response. The role of IFN-γ in HAT progression has been investigated using experimental animal models and in few human studies. In a murine *T. b. brucei* model, IFN-γ knockout mice suffered uncontrolled parasitemia with a significant reduction in survival time compared to the wild type mice [61, 73]. Similar observations were made in a *T. b. rhodesiense* murine model in which IFN-γ was associated with a decrease in parasite numbers and resistance to infection [18]. These findings show that IFN-γ might be essential in parasite control. On the contrary, in a study utilizing mononuclear cell cultures, rat IFN-γ was associated with an increase in parasite numbers [74]. Furthermore, in a murine *T. b. brucei* model an exponential increase in the severity of the neurological response was associated with increased levels of brain IFN-γ [15]. IFN-γ was further demonstrated to be a prerequisite for *T. b. brucei* parasite transmigration across the blood brain barrier (BBB) [75]. In a *T. b. rhodesiense* vervet monkey model, serum IFN-γ was upregulated in the infected group with a positive correlation to body temperature during the early phase of disease. In this model, IFN-γ was never detected in CSF up to day 42 post-infection [60]. Similarly, in human patients, serum IFN-γ was significantly elevated in early stage patients compared to late stage patients and for both stages higher than control levels [71]. In another related *T. b. rhodesiense* sleeping sickness study comparing CSF cytokine levels between two geographically similar HAT foci (Soroti and Tororo), elevated IFN-γ levels were associated with moderate to severe comma [13]. This study provided for the first time, clinical evidence that IFN-γ might be associated with clinical signs of neurological involvement in *T. b. rhodesiense* HAT. In a related study in Eastern Uganda, when CSF IFN-γ concentrations were compared across disease stage no significant differences were noted [76]. Moreover, in the latter study no significant association was noted regarding presence or absence of neuropathology with IFN-γ levels. Within the current literature, the role of IFN-γ in *T. b. rhodesiense* disease remains uncertain.

## Interleukin-1 beta (IL-1β)

Interleukin-1 beta is a pro-inflammatory cytokine belonging to IL-1 family of cytokines [77]. It is produced mainly by monocytes and macrophages [78]. In a number of inflammatory disorders, IL-1β has been associated with both innate and adaptive immune responses [79, 80], with a possible role in BBB dysfunction [81]. However, its role in HAT, is not well defined. In a murine model utilizing a *T. b. brucei* cloned stabilate (GVR35/C1.8), there was an apparent increase in plasma levels of IL-1β but not significantly different from controls [15]. In this study, IL-1β levels were not correlated to the degree of neuro-inflammation. However, in a stepwise multiple linear regression analysis, it was noted that IL-1β, TNF-α and IFN-γ levels in the brain accounted for over 94.8 % of the variation in neuropathology. Furthermore, intraventricular injection of an IL-1 receptor antagonist together with sTNF-r1 antagonist augment the reduction in neurodegeneration caused by trypanosome infection compared with infusion of sTNF-r1 antagonist alone [82]. In a *T. b. brucei* murine model, mRNA transcripts for IL-1β were localized in areas showing apoptosis and nerve fiber degeneration [83]. In this study, neuropathology was not solely attributed to IL-1β. A study involving *T. b. rhodesiense* HAT patients in two Ugandan foci (Tororo and Soroti) reported no significant differences in plasma IL-1β levels despite variations in disease progression and severity between the two foci [13]. In this study, the authors were unsuccessful in detecting IL-1β in the CSF. Available literature does not clearly define the role played by IL-1β in HAT pathogenesis.

## Transforming growth factor-beta (TGF-β)

Transforming growth factor- beta is a pluripotent cytokine with both pro- and counter-inflammatory effects depending on its environment and concentration [84]. At higher concentrations, TGF-β is reported to play an immuno-modulatory role through the suppression of TNF-α and IFN-γ synthesis by peripheral blood mononuclear cells and peritoneal-derived macrophages [85]. In HAT, literature on the role of TGF-β in disease progression is scanty. Nevertheless, TGF-β has been proposed to influence pathogenesis of *T. b. rhodesiense* sleeping sickness. A study comparing *T. b. rhodesiense* cytokine profiles in two geographically distinct HAT foci (Uganda and Malawi) reported a significant increase in plasma TGF-β levels in Malawi patients as compared to patients in Uganda [8]. The authors argued that the higher levels of TGF-β in plasma of Malawi patients might be responsible for the reduced pathology and prolonged survival in this group. On the contrary, Maclean et al. [13] comparing plasma TGF-β levels in two HAT foci in Uganda did not find significant difference despite the disease being more severe in Tororo compared to Soroti. In this study plasma TGF-β levels were significantly higher in HAT patients than controls. These findings suggest an involvement of TGF-β in HAT pathogenesis though its specific role is not clearly understood. In experimental animal models, the role of TGF-β in trypanosomiasis has not been investigated making results from human studies difficult to interpret. Animal models might be helpful in refining the general observations made in human studies.

## Interleukin-6 (IL-6)

Interleukin-6 is a multi-functional cytokine shown to possess both pro- and counter-inflammatory effects with varied implications in pathophysiology of many neurological and inflammatory disorders. In other disorders, IL-6 was shown to possess beneficial effects involving metabolic control [86], neuronal survival [87], neuro-protective and analgesic effects in rats [88]. On the other hand, destructive properties have also been reported. IL-6 has been associated with neuronal degeneration and cell death in degenerative disorders [89]. Furthermore, in other neuropathological disorders, mice over expressing IL-6 were associated with increased BBB permeability coupled with neuropathological abnormalities [90]. In a murine *T. b. brucei* model, high levels of IL-6 were observed in mice with less severe neuropathology [15]. These findings were consistent with studies in a *T. b. rhodesiense* vervet monkey model in which CSF IL-6 levels were up regulated in late stage disease [91]. Similarly, in HAT patients CSF IL-6 was upregulated in late stage *T. b. rhodesiense* disease [76, 92] and in *T. b. gambiense* disease [72, 93]. However, in all these studies, the role of IL-6 in HAT pathogenesis was not investigated. A study comparing plasma levels of IL-6 in two HAT foci in Uganda reported higher levels in Soroti with mild disease as compared to Tororo with a more acute disease [13]. The implication for the elevated IL-6 levels in Soroti patients were not explained. However, it is possible that IL-6 plays a protective role as reported in experimental animals. Consequently, although murine models point to a protective role, in humans the role of IL-6 cannot yet be clearly defined.

## Interleukin-10 (IL-10)

IL-10 is a regulatory cytokine that is produced presumably to control excessive inflammation by a variety of cell types within the innate and adaptive immune systems including macrophages, T- and B-cells [94]. IL-10 has been demonstrated to upregulate the production of antibodies and elevate MHC class II expression on B cells [95]. In a number of parasitic diseases, IL-10 has been shown to possess host protective roles, including malaria [96], toxoplasmosis and in autoimmune encephalitis [97]. Similar roles of IL10 have been described in *T. brucei* experimental murine models.

In one study, the absence of IL-10 in wild type mice was associated with decreased survival in *T. b. brucei* infected mice [61]. Furthermore, mice with increased IL-10 levels were associated with markedly reduced IFN-γ concentrations and subsequently survived longer than infected control animals. The authors thus suggested that IL-10 might play a role in providing a balance between pathogenic and protective immune response during *T. b. brucei* infection [61]. Furthermore, in another murine model using *T. b. brucei* cloned stabilate, mice with elevated CNS IL-10 levels were associated with mild inflammatory pathology [15]. In HAT, plasma and CNS levels of IL-10 were upregulated particularly in the late stage of both *T. b. rhodesiense* [8, 71] and in *T. b. gambiense* [72, 93]. However, in all these studies the role of IL-10 on HAT associated pathology was not investigated. In a study by Maclean et al. [76] among *T. b. rhodesiense* patients in Uganda, CSF IL-10 did not significantly associate with neurological signs of ataxia, tremors, or urinary incontinence. Likewise, a related study comparing plasma cytokine profiles in two foci in Uganda (Tororo and Soroti) did not find a significant difference in IL-10 levels despite the disease being more severe in Tororo. Notwithstanding, even if *T. b. rhodesiense* sleeping sickness has been associated with dysregulation in IL-10 levels, its significance in disease pathogenesis has not been clearly demonstrated.

## Cytokines as potential stage biomarkers

According to a biomarker working group [98], a biomarker is an objectively measured and evaluated characteristic indicating a physiological process, pathogenic process or pharmacological response to a therapeutic intervention. The current HAT staging criteria rely on WBC counting and the detection of parasites in CSF. However, in some cases these criteria have been shown to give false results since trypanosomes are not usually detected in CSF and an elevation in white blood cells is not necessarily specific to trypanosomes [99, 100]. Furthermore, the need for using a lumber puncture to obtain CSF is invasive, a discomfort to the patient and requires trained personnel. Due to these shortfalls in the current staging, a number of novel biomarkers are currently being sought. With the observation that specific cytokines are upregulated in late stage sleeping sickness, they have been proposed as potential stage biomarkers [76, 91]. Among the cytokines, IL-10 and IL-6 have shown greater potential due to their association with late stage disease in the CNS. Additionally, both plasma and CSF IL-10 levels were reported to return to normal following treatment in a *T. b. rhodesiense* vervet monkey model [56], in *T. b. gambiense* sleeping sickness [101] and in *T. b. rhodesiense* sleeping sickness [71]. From these finding, IL-10 was proposed as a marker for cure. However, despite these promising observations, data from clinical evaluation of these markers is limited. In a *T. b. rhodesiense* study, CSF IL-10 was upregulated in late stage patients [76]. When its utility as a potential late stage marker was evaluated, CSF IL-10 was insufficiently sensitive detecting only 14 out of 100 late stage patients [76]. In a more recent study, a higher staging accuracy for both CSF IL-6 and IL-10 was reported [102]. From this study, at a specificity of 100 %, IL-10 would detect around 86 out of 100 late stage patients while IL-6 detected around 83 out of 100 late stage patients. However, in this study the authors acknowledged the fact that most patients were diagnosed as late stage hence limiting comparisons with the few early stage patients. Tiberti et al. [100] demonstrated that when *T. b. rhodesiense* biomarkers are used as a panel, sensitivity and specificity is greatly enhanced. A number of other promising markers distinguishing between early and late stage patients have been proposed including; chemokines and the heart-fatty acid binding protein [41, 100, 103], immunoglobulins [56, 101], cell adhesion molecules [104], neopterin [105], osteopontin and β2 microglobulin [106] and matrix metalloproteinase-9 [104] and recently neuronal specific enolase [107]. Indeed, when CSF IL-10 was evaluated as a panel with TGF-β and IgM, the panel had an improved sensitivity, from detecting 14 out of 100 late stage patients to detecting 70 out of 100 late stage cases [76]. However, to date literature about the use of cytokines as panels to improve staging accuracy is scanty and therefore does not allow meaningful comparisons.

Basing on available literature, the possibility of translating cytokines into point-of-care tests for stage determination has some draw backs. Firstly, cytokine markers are not 100 % sensitive and their application might lead to wrong treatment choices that could lead to relapses since some late stage patients would be missed. In order to improve on sensitivity, studies analyzing cytokines as panels or in combination with other previously identified markers might be helpful. Secondly, the need to rely on the invasive collection of CSF by lumber puncture makes the direct field application of CSF cytokines and other novel markers problematic. To date no plasma cytokine has shown potential as a stage biomarker. To better assess the value of plasma cytokines, studies utilizing larger sample size representative of the population at risk and tools such as Luminex Chips that enable the quantification in a single sample of large numbers of cytokines or proteomic approaches such as the one performed on the CSF are required. Lastly, sleeping sickness is endemic in areas were other tropical diseases are common [7]. This is complicated by the fact that cytokine dysregulations and biomarker potential apply to other CNS disorders [108], thus clouding direct interpretation of cytokine data. Therefore, clinical validation of cytokine data in light of other co-infections would be helpful in identifying specific cytokines that might be unique to HAT.

## Conclusions

Although literature on cytokine dysregulation in *T. b. rhodesiense* HAT is scarce, it is quite clear that high levels of pro-inflammatory cytokines are associated with immunopathology. However, in late stage disease, an elevation in counter-inflammatory cytokines is associated with a reduction in the degree of immunopathology. In some cases, inconsistences about the role of specific cytokines in HAT pathogenesis have been documented. This points to the fact that pathogenesis might be influenced by a complex interaction of cytokines with many having multiple roles. Moreover, due to ethical considerations in human studies, serial measurements of cytokines with disease progress have not been done making a clear distinction between cause and effect roles for the specific cytokines problematic. Nevertheless, cytokines have been proposed as potential diagnostic or stage biomarkers. Indeed, due to their up-regulation in late stage diseases, counter-inflammatory cytokines including IL-10, IL-6 and TGF-β were proposed as stage biomarkers. However, before cytokines can be considered as biomarkers, more clinical studies are required for validation. On the other hand, though murine models have provided invaluable information, inconsistences from human studies have been reported. Therefore, extrapolating data from these models might give erroneous conclusions about cytokine roles in HAT. Recently, the vervet monkey model has been shown to develop disease clinically and immunologically similar to *T. b. rhodesiense* HAT in humans [91] and would be helpful in refining cytokine roles with disease progression.

## Abbreviations

BBB: blood brain barrier
CNS: central nervous system
CSF: cerebrospinal fluid
HAT: human African trypanosomiasis
IFN: interferon
IL: interleukin
LAMP: loop mediated isothermal amplification
MGE: mobile genetic element
mHCT: mini-anion-exchange centrifugation technique
PCR: polymerase Chain Reaction
RAPD: random amplified polymorphism DNA
RDTs: rapid diagnostic tests
SRA: serum resistance antigen
TGF-β: transforming growth factor- beta
TNF: tumor necrosis factor
WBC: white blood cell
WHO: World Health Organization
